# Effectiveness of bicycle helmets and injury prevention: a systematic review of meta-analyses

**DOI:** 10.1038/s41598-023-35728-x

**Published:** 2023-05-26

**Authors:** Carlson Moses Büth, Natalia Barbour, Mohamed Abdel-Aty

**Affiliations:** 1 Department of Physics, University of Münster, 48149 Münster, Germany; 2 Department of Civil, Environmental and Construction Engineering, University of Central Florida, Orlando, FL 32816 USA

**Keywords:** Medical research, Engineering

## Abstract

To mitigate the risk of injuries, many countries recommend bicycle helmets. The current paper seeks to examine the effectiveness of bicycle helmets by performing a systematic review focusing on meta-analyses. First, the current paper explores the findings of studies that employ meta-analyses using bicycle crash data. Second, the results are discussed considering the findings from research analyzing bicycle helmet effectiveness in a laboratory using simulation, and then are complemented with key methodological papers that address cycling and the overall factors contributing to the injury severity. The examined literature confirms that wearing a helmet while cycling is beneficial, regardless of age, crash severity, or crash type. The relative benefit is found to be higher in high-risk situations and when cycling on shared roads and particularly preventing severe head injuries. The results from the studies performed in laboratories also suggest that the shape and size of the head itself play a role in the protective effects of helmets. However, concerns regarding the equitability of the test conditions were found as all reviewed studies used a fifty-percentile male head and body forms. Lastly, the paper discusses the literature findings in a broader societal context.

## Introduction

Cycling safety has been a focal point of many discussions relating to transportation, sustainability, and public health. The shift in environmental awareness, historical auto-dependance and sedentary lifestyles have encouraged many people to use bicycles as a mode of transport as opposed to recreation^1^. Although cyclists generally do not travel at high speeds, unsafe intersections and lack of designated cycling infrastructure have led to a worrying number of injuries. A total of 2035 cyclists died in Europe in 2019. Furthermore, the relative proportion of seriously injured cyclists also increased from 7 percent in 2010 to 9 percent in 2019^2^. The same is true for the United States, where 938 cyclist fatalities were registered in 2020 and that has been the highest number since 2011. Furthermore, a total of 38,886 serious injuries were reported in the US^3^. These numbers are likely higher since cyclists’ injuries are still considerably under-reported, particularly in cases where no automobile was involved^4^.

The objective of this study concerns bicycle helmets and aims to provide a multi-perspective view of the protective effects of bicycle helmets and their effectiveness. To achieve this goal, a systematic review of select studies that focus on helmet effectiveness from various approaches, emphasizing observational studies, was conducted. Systematically selected publications are presented, and their results discussed in a structured manner to provide a cohesive review of studies relating to the bicycle helmet effectiveness.

Specifically, the issue of the risk compensation hypothesis that argues increased risk-taking when cyclists use helmets will not be addressed. There has already been an extensive peer-reviewed literature review conducted by Esmaeilikia et al.^5^, which found little to no support for increased risk-taking when cyclists use helmets and if anything, they cycled with more caution.

Because the helmet usage, culture, and crashes relating to cycling vary per location, to ensure the most accurate and broad analysis, the current approach combines the findings from multiple meta-analysis studies. The paper begins with a comprehensive but brief description of different study designs and tools that are most frequently used to study this topic and is followed by the description of the literature selection for the purpose of the study. The findings of the meta-analyses using bicycle crash data are then presented and complemented with laboratory based and simulation safety studies and followed by the key papers addressing cycling and the overall factors contributing to the injury severities. The paper concludes with the discussion of findings.

### Types of studies

Assessing the effectiveness of an intervention has no single definitive measure, however, there are two main observational study designs often applied to investigate the effectiveness of helmets on head injuries. Randomized controlled trials are not a viable approach because a bicycle crash is an undesirable event, which makes this approach unethical.

### Case–control studies

The majority of investigations on helmet effectiveness are case-control studies. This design is suitable for studying undesirable events. Explicitly, they compare the odds of an injury in the groups of cyclists wearing helmets with those who are not wearing helmets.

By collecting the information on cyclists wearing a helmet during the crash, its location, possible injury and its severity, the odds ratio (OR) can be calculated (Table 1). An odds ratio (OR) is a measure of association between an exposure and an outcome^6^. For helmet-wearing cyclists, the odds of injury are a/b; for cyclists not wearing helmets, the odds are c/d. The odds ratio is the ratio of these two odds. Calculated OR smaller than 1 means that wearing a helmet is associated with lower odds of outcome (injury). Relative risk (RR) describes another measure of the risk that studies a certain event happening in one group compared to the risk of the same event happening in another group^7^. Furthermore, a log relative risk variance can be calculated to obtain a confidence interval (CI) and to do a statistical hypothesis test. When the upper CI is also below 1, the outcome becomes statistically significant. Case-control studies cannot produce RRs with their data, however they can offer ORs, which is why they are often reported^8^.

**Table 1:** Contingency table with odds ratio $OR=\frac{a/b}{c/d}=\frac{ad}{bc}$ where *case* means injured cyclists and *control* stands for not injured^8^.

	Case	Control
Helmet	a	b
No helmet	c	d

### Cohort study designs

Somewhat less popular than case–control studies in exploring helmet effectiveness are cohort studies. In a cohort study, the study population is selected on the basis of exposure (e.g., wearing a helmet) as the case group, then followed over time and compared with an unexposed control group. Depending on the data availability, the RRs or ORs are still determined. If the number of cyclists wearing helmets (${n}_{h}$) and not wearing helmets (${n}_{nh}$) is known, the relative risk is RR can be calculated $RR=\frac{a/{n}_{h}}{c/{n}_{nh}}$ (where $a$ and $c$ have been previously defined). One of the major drawbacks of this measure is the need to know the total number of cyclists to determine CIs, which is very difficult to obtain. Consequently, it means that hypothesis testing is not possible. Because a very large-scale study is needed, cohort study designs have not been as widely used as the case-control study^9^. Furthermore, although the case-control studies use data more efficiently and might be more reliable because the data are collected in conjunction with injury, cohort study designs could provide additional insights into the effectiveness of a studied measure.

### Before and after studies

The simplest design assessing temporal changes is a before and after study. Before and after studies measure outcomes in a group of cyclists before introducing an intervention (e.g., mandatory helmet policy), and then again afterwards. A variation of this is the interrupted time series design, where the data before and after the intervention are time series. One way to evaluate the effectiveness is to study the numbers of injured cyclists and compare the findings between the before and after the mandatory bicycle helmet legislation is introduced. Investigation of the effect takes place over time in a more aggregate manner as opposed to comparing individual crashes of cyclists with and without helmets^10^. One instance of such study by Olivier et al.^11^ showed that there was a 46% reduction in fatal crashes after the introduction of the bicycle helmet law in Australia.

### Meta-analysis

Because many studies are performed in a constrained geographical location with specific environmental characteristics, the results of individual studies cannot be considered generally applicable to other contexts. To improve precision, broaden the applicability of the findings, and answer questions on a higher spatial level, the results of multiple studies can be combined to form a meta-analysis. This approach is typically used in systematic reviews in medical fields. Especially in the case of contradictory claims, this method can provide clarifications that would be difficult to provide otherwise. A standard in the field of medicine is the Cochrane Handbook for Systematic Reviews of Interventions^12^, which is updated regularly.

In the case of undesirable events such as bicycle crash injuries, it is likely that single studies with limited data may not be able to assess the effectiveness of helmets in different contexts and conditions. Meta-analyses combine multiple data points to deliver in-depth conclusions on whether an intervention has an impact on undesirable incident occurrence such as the crash involving a bicycle. Combining multiple studies into one meta-analysis allows to mirror the complexity of reality more accurately.

### Data and papers selection methods

To get a systematic review of the literature, a search in the most relevant transport journals has been performed. Relevant articles from journals ranked Q1 (n = 15) and Q2 (n = 17) were identified using the SCImago Journal Rank in the category of transportation. Additional journal sources were identified through the Transport Research International Documentation (TRID) database (n = 4). The search to screen peer-review literature was performed in March 2022 as a single screening. Using Google Scholar, the selected journals were then searched by the keywords: safety, helmet, exposure, and bicycle or bike. Literature selection aimed to identify studies that met the criteria of being current or conducted on a large scale. Explicitly, this means that the focus fell primarily on recently published articles and excluded publications prior to 2015, except those distinguished by being conducted on a large scale, namely meta-analyses. Some articles that were not relevant were excluded based on the following eligibility criteria. Generally, the literature was excluded when the primary focus did not include helmet effectiveness, or when the articles had been retracted, superseded, or represented opinions. Additionally, to determine a clear focus of the study, three perspectives on bicycle helmet effectiveness are evaluated. The first one includes primarily observational studies based on the effect of bicycle helmets on cyclists’ injuries involved in a crash. The second perspective examined research that did not require a human component and the studies were conducted in a laboratory setting using simulation. Lastly, the third one addressed general injury severity factors that contextualize the use of helmets. Because a comprehensive review of the topics associated with helmet legislation was already provided by Olivier et al.^13^, the current study aims to primarily examine the helmet effectiveness as opposed to investigate helmet related policies and laws. To further confirm the selection of the articles, a second-degree citation network search using Litmaps 2021 was performed. Litmaps 2021 internally sources data from Microsoft Academic Graph and Semantic Scholar to build the citation network based on the articles’ eligibility.

**Figure 1:**
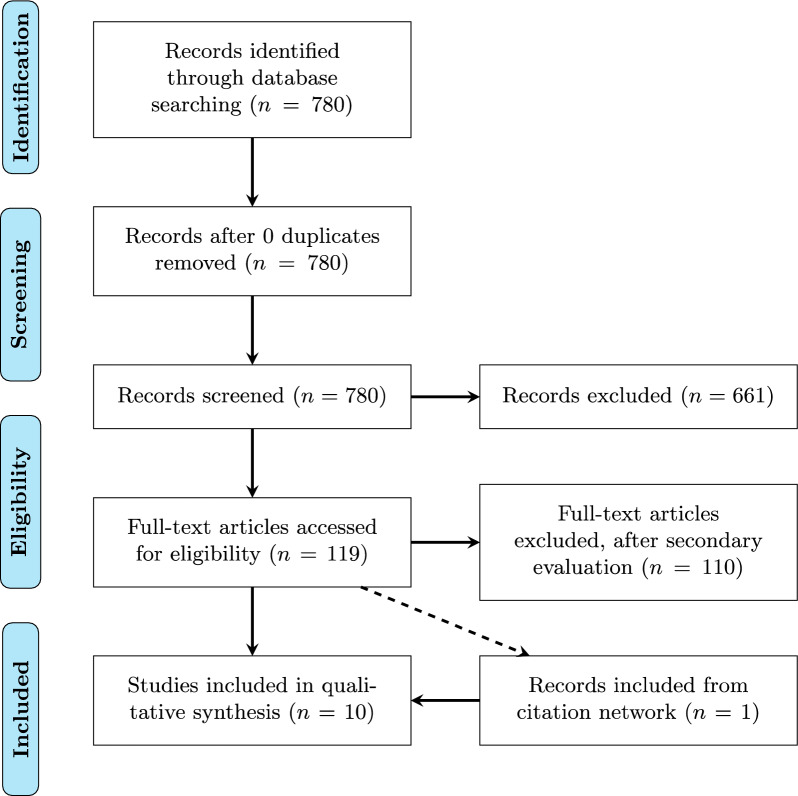
Flow diagram of included studies, based on the Preferred Reporting Items for Systematic Reviews and Meta-Analyses—PRISMA^14^.

A total of 780 records were identified (Fig. 1). Each journal was only searched once, therefore there were no duplicates to eliminate. All records were then subjected to a secondary screening and 119 papers have been selected for further evaluation based on the abovementioned criteria. Lastly, due to the use of citation mapping technique (Litmaps), a selection of 10 key articles was made. The first group yielded five articles and included systematic reviews and meta-analyses that studied helmet effectiveness based on observational bicycle crash data. In addition, it included the most recent meta-analysis involving intervention, specifically mandatory helmet laws. For laboratory and simulation, three studies were found, and finally the third group includes two key studies.

### Bicycle crash data studies

Academic research produced only a handful of meta-studies aiming to explore bicycle helmet effectiveness. The earliest one, which includes seven study papers, is a Cochrane Review published by Thompson et al. in 1999^15^. Attewell et al.^16^ added nine more review papers to the former review, followed by Elvik who expanded it with four more studies, which summed up to twenty papers in total^17,18^. Most recent and complete studies on helmet effectiveness were published by Olivier and Creighton^19^ as well as Høye^20^. Around the same time, Olivier and Radun^8^ provided a more in-depth justification of used methods validity by examining their strengths and weaknesses. Finally, Høye^21^ analyzed the results of studies on mandatory helmet legislation. We exclude the earliest review by Thompson et al.^15^ because four of the included studies were performed by the authors themselves and were included in other meta-studies that were indeed incorporated in the current paper, as well as Elvik^17,18^ because the authors only analyzed a few more studies than Attewell et al.^16^ and these few studies were later investigated by Olivier and Creighton^19^ in more detail.

The study selection process was a systematic literature search to establish and justify their general validity and usability in the current study. The aim of Attewell et al.^16^ was to quantify the efficacy of helmet use in preventing serious injuries to cyclists. The authors searched the Medline database with the keywords such as “bicycle helmet”, “efficacy” and “head injury”. Inclusion criteria for the literature had to be written in English, peer-reviewed, and have individual cyclist data leading to a 2 × 2 table of injury by helmet use, as required by the case–control studies. Two reviewers then performed an independent search to confirm those studies and discussed the reasons behind including or rejecting a particular study. Only 16 of 63 included papers were eligible for numerical analysis, the rest 47 had an incompatible design, other inadequacy or added no new data. Still, most of the U.S. studies in Attewell et al.^16^ came from Thompson et al.^15^. Five different injury type groups by body region and severity were identified and analyzed. Olivier and Creighton^19^, on the other hand, searched through Medline, Embase, Compendex and Scopus with the keywords “helmet*” and “cycl*” or “bicycle*”, where * indicates any word ending. So “bicycle*” matches “bicycle” as well as “bicycles”. Additionally, the crashes that indicated injury severity had to be medically diagnosed and excluded self-reports. PubMed, Sciencedirect, and Google Scholar were the databases for the meta-analysis of Høye^20^, which used the searches with keywords “bicycl*” or “cycl*” and “helmet”, combined with either “injur*”, or “fatal*”. Similar inclusion criteria that allowed ORs of any subgroup to be calculated, as well as statistical weights based on the significance, confidence interval, t-value or the number of observations. Olivier and Radun^8^ reviewed the methodological challenges estimating bicycle helmet effectiveness and did not claim to draft a systematic report. They reanalyzed the data from Zeegers^22^ and added some additional data from a Dutch study to point out possible pitfalls while estimating helmet effectiveness with respect to case-control and cohort study designs. Finally, concerning mandatory helmet legislation Høye^21^ used PubMed, Sciencedirect, and Google Scholar with expanded search words: “bicycl*” or “cycl*”, “helmet”, “injur*” or “fatal*”, and “legislation” or “law”. The criteria were similar to their former work^20^, but only included studies that had comparison or controlled groups for confounding variables. This excluded the studies where the authors only gave simple before and after injury rates and the papers that did not provide absolute numbers.

Table 2 presents the details of the papers that were included in the current review. The research by Elvik^17,18^ is not included because it only took into consideration a handful more studies than Attewell et al.^16^, which were addressed in detail by Olivier and Creighton^19^. Five of the selected studies were included in the analysis and are presented in Table 2. The second group of included research papers (three studies) examine the effectiveness of bicycle helmets in the laboratory setting using simulation, and finally the third group includes two key studies about general injury severity factors that contextualize the use of helmets.

**Table 2:** Meta-analyses bicycle crash studies on bicycle helmet effectiveness and preventing injuries in different body regions.

Reference	Studies included in the analysis publication date range	Number of studies included	Countries evaluated	Study designs	Aim	Results (ORs and 95% CI)	Conclusions
Attewell et al.^16^	1987–1998	16	USA (9), Australia (4), Canada (2), UK (1)	Case control (OR, 95% CI)	Quantification of helmet use efficacy in preventing serious cyclist injury.	Head: 0.40 (0.29–0.55) Brain: 0.42 (0.26–0.67) Face: 0.53 (0.39–0.73) Neck: 1.36 (1.00–1.86) Fatal: 0.27 (0.10–0.71)	Clear evidence for helmet use risk reduction of head, brain, facial and fatal injury. For all ages and in single bicycle or motor vehicle crashes. No clear sign of publication bias.
Olivier and Creighton^19^	1989–2015	40	USA (17), Australia (9), UK (1), Canada (4), Norway (1), Singapore (1), Finland (1), Hong Kong (1), France (1), Germany (2), Sweden (2)	Case–control (OR, 95% CI) using multi-variate meta- regression model	Whether helmet use mitigates head, serious head, face, neck and fatal head injury. Test for time trend bias.	Head: 0.49 (0.42–0.57) Serious head: 0.31 (0.25–0.37) Fatal head: 0.35 (0.14–0.88) Face: 0.67 (0.56–0.81) Neck: 0.96 (0.74–1.25)	Helmet use reduces injury odds ratios, only neck injuries are not associated with it. Safety strategies should consider uptake of bicycle helmets. No clear sign of publication bias nor time trends.
Høye^20^	1989–2017	55	USA (22), Australia (12), UK (1), Canada (5), Germany (6), Norway (1), Singapore (1), Finland (1), Hong Kong (1), France (1), Sweden (2), Taiwan (1)	Case–control (OR, 95% CI), T&F, comparison between subgroups	Replicate and extend Olivier and Creighton^19^ and investigate additional variables.	Any: 0.53 (0.37–0.76) Head: 0.47 (0.36–0.61) Serious head: 0.40 (0.35–0.46) Fatal head: 0.29 (0.15–0.56) Face: 0.77 (0.67–0.89) Cervical spine: 1.09 (0.90–1.32)	Despite methodological differences (un-/adjusted, hospital/police data, adult/children) meta-analyses show helmet use reduces head injury, specifically serious and fatal head injuries.
Olivier and Radun^8^	1989–2013	Not stated	USA (Seattle Victoria), Australia (New South Wales), Netherlands	Case–control (OR, 95% CI; RR)	Discuss challenges in estimating bicycle helmet effectiveness and reanalyze data used by Zeegers^22.^	Results favorable of helmet effectiveness compared to Zeegers^22^ where reported data was in conflict with source material.	Case-control studies cannot estimate the probability of a crash or fall, but their results are the best available evidence. They suggest helmet use is an effective measure in reducing head injuries.
Høye^21^	1994 –2018	21	Australia (5), USA (8), New Zealand (1), Canada (6), Sweden (1)	Before and after case control	Summarize safety effects of mandatory bicycle helmet legislation on head injuries.	Injuries in general and especially serious head injuries are reduced with the legislation. It is more effective for everyone if it applies to everyone, not only to children.	Empirically, mandatory bicycle helmet legislation is an effective measure to reduce serious head injuries in crashes involved cyclists.

## Results

The results section is divided into three main parts. First, the findings from the bicycle crash data studies are presented, followed by the findings from the laboratory and simulation studies, and third the results of the injury severity and cycling studies are discussed. The first section where the bicycle crash data studies are investigated is further divided into the following subsections: bicycle crash data studies: publication bias, bicycle crash data studies: cyclist behavior, bicycle crash data studies—final remarks.

### Bicycle crash data studies: findings on helmet effectiveness

Findings on helmet effectiveness concerning different body regions (such as neck or face) that were found by the three meta-studies with their respective 95% CI are presented in Table 2. Interestingly, as a disclaimer, Olivier and Creighton^19^ pointed out that the investigation of exactly one injury type is not as straightforward, because most injuries come paired with others. They emphasize that police report data might document a more severe Traumatic Brain Injury (TBI) while ignoring a minor facial injury. If multiple types have been documented, the more severe one generally determines the overall injury severity category.

Studies included in the meta study of Attewell et al.^16^ vary in size from 21 to 3390 cases and include a variety of injury types. All age groups were represented with children being overrepresented. Head injuries were found to be reduced significantly with helmet usage by 60 percent, brain injuries by 58 percent and facial injuries were reduced by 4 percent. The impact on neck injuries was shown to be insignificant. Fatal injuries were shown to decrease significantly by a prominent 73 percent if the cyclist was wearing a helmet. Only the subgroup of children resulted in a higher injury rate, which might be due to hospital admission as an inclusion criterion. A broader and more recent study that was based on the crash data suggested that for children helmet wearing decreases the risk of severe injury^23^. Attewell et al.^16^ concluded that wearing a helmet reduces the overall risk of an injury, even at conservative upper confidence intervals. Only seven of the total sixty-three articles that Attewell et al.^16^ included in their research did not endorse helmets.

Olivier and Creighton^19^ included a larger number of studies, but excluded self-report and data published in abstracts only. They found a significant reduction in all their injury groups, especially severe injuries. Except for the impacts on neck injury, which yielded near to a null effect. Olivier and Creighton^19^ stressed out that the magnitude of injury reduction turned out to be higher in serious injuries compared to groups with any injury severity. Serious head (69 percent) and fatal head (65 percent) injuries saw a clear reduction in severity.

Høye^20^ included 55 records in the analysis (including abstract exclusive data) and studied a slightly different injury group. The research included a summary group of any injury type and any severity, but instead of a neck they considered cervical spine injury. Again, all but one group showed a significant decrease in OR, a decreased risk of injury while wearing a cycling helmet, mostly for a fatal head injury. Compared to the results obtained by Olivier and Creighton^19^, the resulting ORs are similar. ORs of fatal head injury and unspecified severity are even slightly smaller but insignificant. The serious head OR is 9 percentage points higher and the face injury group is 10 percentage points higher with both being statistically significant. Høye^20^ expected varying types of facial accidents to be affected in varying magnitudes but the only group with an OR above 1 was the cervical spine group. As expected, wearing a helmet during a crash does not significantly decrease such injury, but neither amplifies it.

When examining the safety effects of a mandatory bicycle helmet legislation with case-control and before-after study designs, Høye^21^ found that such legislation led to a reduction in injuries. Again, especially serious injuries were decreased. There were two interesting findings; first, the legislation only applying to children does only not significantly reduce serious injury among them, but also among the adults and second, the effects are even greater for all age groups when the mandatory helmet legislation is issued without age differentiation.

#### Bicycle crash data studies: publication bias

All discussed meta-studies addressed to varying degrees publication bias (PB) as a possible limitation. Publication bias describes that possibly studies with statistically significant results are more likely to be published. A visual cue are funnel plots, which plot standard error against its estimate, which should be symmetrical around the average and narrow at the top. Publication bias can be one reason for skewness of the funnel, which has been used as an indicator (for details and example please refer to Attewell et al.^16^). Also, Elvik^17,18^ inferred publication bias from this ten years later and addressed it using the trim-and-fill (T&F) method that argues to make the funnel plots symmetric before taking the final estimate. Olivier and Creighton^19^ stressed that applying the trim-and-fill technique may lead to underestimating of ORs if publication bias does not exist. Additionally, Olivier and Creighton^19^ used formal criteria such as rank correlation test to determine publication bias. No strong evidence of publication test was found (τ = − 0.08, *P* = 0.25), which made the need of the trim-and-fill obsolete in the first place. Høye^20,21^ used funnel plots and corrections by applying the trim-and-fill method where the funnel plots were asymmetric, which reduced the effect of publication bias. It is important to point out that the symmetrization of the funnel plot does not lead to the elimination of publication bias, since such skewness may have other causes.

#### Bicycle crash data studies: cyclist behavior

Cyclist behavior and particularly shift in risk taking in response to wearing a helmet was found to be an important point of consideration in the reviewed studies. To study this phenomenon, Olivier and Creighton^19^ made an adjustment for effects that might be induced by a theoretical risk compensation, and found the adjusted odds to be nearly identical, suggesting that such effect does not exist. The decrease of fatalities or serious injuries (KSI) in cyclist groups likely outweighs any effect of behavioral adaptation that wearing a helmet might have. Nevertheless, it remains unclear if behavioral effects are positive or negative on injuries^20^.

When comparing studies in areas with and without a mandatory bicycle helmet legislation, a tendency towards greater protective effects on head injuries when use is mandatory are generally shown^21^. After an introduction of a mandatory helmet legislation, a decrease in cyclists might occur, but such effect did not necessarily last for a long time. Surveys showed that other factors are much more important in making cycling a valid or invalid option for the general population. Høye^21^ argued that a possible self-selection effect would decrease the average crash risk in general, due to a variety of characteristics cyclists who decide to wear a helmet have and often exhibit a safer cycling behavior in general. Studies on behavior adaptation found no clear causality between helmet use and risk-taking behavior^21^.

#### Bicycle crash data studies: final remarks

While all the reviewed studies agree that helmet usage protects against head injuries, most studies also emphasize that helmets are most effective in preventing serious and fatal injuries. Also, the effects of bicycle helmets are larger in single bicycle crashes^20^. Attewell et al.^16^ concluded that their result of increased neck injury should continue to be monitored and might be related to the helmet type. Other studies that explored this phenomenon did not reproduce this finding. Wearing a helmet while cycling has been found to have a clear benefit on injury reduction, for all ages, independent of severity, and in bike crashes that may or may not involve a motorized vehicle.

### Lab and simulation studies

To test the effectiveness of bicycle helmets without risking the well-being and health of cyclists, some studies investigated their protective capability in the laboratories. Studies employing simulation allow for standardization of test conditions and do need the real-world environment to collect the data. Each helmet that is approved on the market must comply with local laws and therefore needs to be subjected to testing in consistent conditions. In Europe, the regulations are guarded by En 1078:2012 (En1078 2012), in the USA by CPSC 16 CFR 1203-08 (16 CFR Part 1203 1998), Japan JIS T 8134:2007, Australia and New Zealand AS/AnZ 2063: 2008, and China applies their GB 24429–2009. All the outlined standards only use the peak linear acceleration (PLA) at the head’s center of gravity^24^. The standards fail to account for oblique impacts, where rotational dynamics exist. The rotational dynamics are known to be the most common scenarios in the real cycling conditions^25^. The literature search yielded three papers on the complexity of this topic. The first identified study by Bland et al.^26^ meant to identify the helmets’ differences in terms of their protective capabilities between the standard lab conditions and the real-world scenarios. The impacts were tested with a standard drop rig of four helmet models and compared the standard specified conditions to the ones commonly found in the real-world environment. To compute further injury criteria Deck et al.^27^ added a rotational acceleration sensor on a head model that represented a more realistic rotational inertia. Using the acceleration data, the study simulated a brain finite element (FE) model to obtain an indicator based on tissue level brain injury criteria, which can predict a moderate diffuse axonal injury (mDAI) such as moderate neurological injuries or short coma. Finally, Wang et al.^24^ aimed to mirror accurately different scenarios using a full-scale multibody (mB) simulation. Digital models of helmet’s effectiveness were created and validated through drop tests before being used in full-scale simulations for a cyclist. Impact scenarios with a cyclist’s head impacting a curb and cyclist skidding were simulated with and without the helmet models for comparison. Wang et al.^24^ made use of further methods of computational biomechanics—nine helmet models were first modeled by laser scanning and the material properties were determined experimentally. By comparing brain injury severities when wearing a helmet versus not wearing a helmet, the helmet head-protection effectiveness was scored. The cyclist model in Wang et al.^24^ was found to be the most complete and featured a head-neck complex of the Total Human model for Safety THUmS finite element model, coupled to the full-scale multibody model pedestrian model (again 50th percentile adult man), sitting on a validated model of a typical road cycle. First, all three studies came to the results that vary significantly depending on the helmet model, nevertheless, some interesting results were found.

Compared with a cyclist not wearing a helmet, the risk of skull fracture decreased across all helmets if cyclist was wearing a helmet^24^. On average, there was an 80 percent reduction in skull fractures for curb-impact and a 65 percent reduction for skidding. When comparing protective capabilities between the standard and real-world scenarios, Bland et al.^26^ showed that the risk of severe brain injury is negligible (0.2 percent to 2 percent) at lower velocity. Four of the ten models (including all non-road-style helmets) were found to have an unacceptably high risk of 88 percent to 97 percent for the temporal impact. In line with these results, Deck et al.^27^ obtained the most critical head injury criterion (HIC) values for the lateral and as well as occipital impacts. With the brain injury criterion (BrIC), they identified oblique impacts causing rotation around the axis represented by the neck to be the most severe. While the skidding impact shows only minor differences, a substantial difference was found between the global kinematics for the curb-impact scenario with and without a helmet. In parity with the consistently reduced global parameters (PLA and HIC based), Wang et al.^24^ examined both and concluded that brain deformation criteria also point to helmets to be protective in every scenario studied, even if the level of protection varied. The maximum principal strain (mPS) was greatly reduced, and cumulative strain damage measure (CSDm) varied by the helmet type.

Protective effectiveness of the bicycle helmets varied from model to model at standard impact velocity, yet this is not apparent to the consumers. The variation in injury risk varies more at the temporal location. Bland et al.^26^ considered a larger radius of curvature, larger contact area, and associated higher stiffness to be the factors contributing to the unfavorable energy absorption resulting in a higher risk of injury. Frontal impacts at the rim below the standardized lines are common, but some helmets fail to provide protection at these points. Optimization only at certain standardized zones might be disadvantageous at other impact points. Bland et al.^26^ concluded the protective effectiveness of cycling helmets to differ between real-world and standard conditions. The researchers point out that the current standards are important and testable areas should be expanded, not replaced. Although rotational acceleration has been known to be relevant in cyclist injuries, it is still missing in standardized testing today. Using full body simulation, Wang et al.^24^ confirmed that rotational acceleration is indeed increased when wearing a helmet. A standard incorporating such criteria could reduce the introduced effect by changing the helmet design. Deck et al.^27^, found no correlation between any of the global kinematic parameters and the simulated model-based brain tissue injury. Even if the effects may vary, helmets’ overall protective effect against injury is confirmed by Wang et al.^24^ in terms of the head injury criterion (HIC) and skull fracture for both curb-impact and skidding impact scenarios.

The results suggest that the shape and size of the head itself also play a key role in the protective effects of bicycle helmets. All three studies used a fifty-percentile male head and body forms. There is no reason to assume that helmets standardized for a specific head shape will be safest for individuals with other anthropometric characteristics. To be equitable, the future standards should not be exclusive to studying average men but should include much broader and more diverse population.

STAR protocol developed by the Virginia Tech Helmet Lab that incorporates the oblique impacts^28^, suggests that the voluntary indication of the protective capabilities provided to consumers should become more common.

Simulation and dummy tests are a powerful tool in assessing the effectiveness of bicycle helmets, but only to some degree, are capable to mirror the reality. Both approaches are qualitatively different. While dummy tests take place physically, simulations approximate laws of nature by computation. Surely, both cannot entirely account for the human nature, but as wisely used tools, they can provide more insights into the protective effectiveness of helmets.

### Injury severity and cycling

To paint a more comprehensive picture and complement the above-mentioned analysis, it is important to examine the findings considering the injury severities among the cyclists. Behnood and Mannering^29^ investigated the severity of crashes between bicycles and motorized vehicles, while Myhrmann et al.^30^ used statistical models to estimate the severity of single-bike crashes by accounting for class-specific heterogeneity.

Research has shown that there are several key factors that can influence the severity of injuries of cyclists. These factors primarily include the characteristics of vehicles and drivers involved, as well as the environmental conditions such as the quality of the infrastructure or weather^31^. Past studies that have partitioned datasets under explanatory variables do not always account for unobserved heterogeneity in the data. Nevertheless, a wide range of models that are capable to account for unobserved heterogeneity have been widely used to handle big datasets with many variables^32^. Using advanced statistical methods, Behnood and Mannering^29^ analyzed the Los Angeles police-reported bicycle-vehicle crash data.

Over a seven-year period, 5,653 crashes were recorded, and one of the three injury categories noted: no visible injury, minor injury, severe injury (including death). For each crash the police data captured variables about the bicyclist’s and driver’s characteristics, their movement preceding the crash, environmental and location-related factors, and other variables such as helmet use. Myhrmann et al.^30^, on the other hand, took hospital’s emergency department data from Aarhus (the second most populous municipality in Denmark) (years recorded 2010–2015, N = 4250 injured bicyclists). Their dataset included information about road victim’s age, gender, helmet use, injury severity, as well as road type, surface condition, time and location of the crash. The authors combined the above-stated dataset with the road maintenance data, and their final dataset included 1,720 single-bicycle crashes with one of the injury severity categories: no injury, slight injury, severe injury.

Since it is not possible to collect all the factors that affect injury severity, it is essential to account for the unobserved heterogeneity in the data. This idea originates from the fact that all the explanatory variables do not account for the full extent of heterogeneity in the conditional mean (and possibly variance) across the dependent variable^29^.

Behnood and Mannering^29^ analyzed the Los Angeles data, allowing for crash-specific unobserved heterogeneity and estimated random parameters multinomial logit model with heterogeneity in the means and variances of random parameters on bicyclist’s injury severity. The model is estimated by simulated maximum likelihood using 1000 Halton draws.

Using the data from the emergency department in Demnark, Myhrmann et al.^30^ choose a latent class ordered probit model, where the injury severity of a cyclist was estimated using the multinomial logit regression (MNL). Model estimation was made using maximum likelihood estimation (MLE). Both studies computed the marginal effects to formulate their findings.

Furthermore, both studies found that there are several factors that contribute to an increased likelihood of serious injury for both—bicycle-vehicle crashes and single-bicycle crashes. Behnood and Mannering^29^ found for police-reported bicycle-vehicle crashes that driver’s race and gender, alcohol use by any party, older age, using the wrong side of the road, speeding driver, not wearing a helmet were found to increase the likelihood of a serious injury. Single-bicycle crashes were more likely to become severe crashes on shared road sections compared to bike lanes as well as on poorly maintained bike lanes or roads with little traffic and after dark. Regarding the helmet usage, not wearing a helmet in the Los Angeles study, was found to decrease the likelihood of a non-visible injury as well as was increase the likelihood of minor and major injuries^29^. The Aarhus hospital’s emergency department data showed no change for severe injury when wearing a helmet but a significant increase in the probability of not being injured and an inverse decrease in the probability of being slightly injured^30^.

Various factors affect the severity of bicycle crashes. Wearing a bicycle helmet was shown, in both studies, to significantly decrease the likelihood of slight/minor injuries and increase the likelihood of no (visible) injury.

## Discussion and conclusions

In this study, a systematic review of papers exploring helmet effectiveness in preventing injury from three structurally different perspectives is presented. The empirical evidence based on the real-world hospital and police data as well as biomechanical studies confirms that wearing a helmet while cycling is beneficial, regardless of age and crash severity, in collisions with others or not. The relative benefit is higher in high-risk situations and when cycling on shared roads. The findings from the meta-analyses studies that have been reviewed in this paper are remarkably consistent.

Given the findings, perhaps more fundamental questions relating to the societal and cultural context and injury prevention need to be addressed. The analyzed literature clearly states that helmets are the most effective in preventing severe head injuries, which are often a result of vehicle-bicyclist crash and while some governments have been promoting practices of safe cycling, the law compliance has historically varied. Valero-Mora et al.^33^ investigated this phenomenon and found that this variation did not seem clearly related to the prevailing bicycle helmet law. The authors found that while in the Netherlands, people knew that helmets were not mandatory and they often reported not wearing them regularly (only 2.4 percent reported always using them), in Norway, under similar conditions about 80 percent reported always wearing a helmet. Consequently Valero-Mora et al.^33^ concluded that though the laws by themselves may not yield a sufficient effect without proper publicity to make riders aware of such laws, awareness-raising campaigns are critical for convincing people to wear helmets. Same authors used their modelling estimation results to indicate that the belief that helmets are mandatory together with the age and gender of the respondent were significant predictors of helmet use, but the helmet law itself did not predict the reported use of bicycle helmets. They also noted that the country where the helmet is most frequently reportedly used (Norway), does not actually have a mandatory law, while other countries with similar absence of helmet laws—Turkey, Greece, Portugal, Sweden, or Israel—report higher rates of helmet use compared to e.g., Argentina, which has such a law. Recent study by Ledesma et al.^34^ investigated the root causes associated with helmet use by adult cyclists and interestingly found that even after controlling for the socio-demographic and cycling-related variables, the belief factors relating to wearing a helmet were among the strongest predictors of their use. The strongest association was linked to the subjective norms as well as the influence of the family and peers and particularly their behavior with respect to wearing helmets^35,36^. Davison et al.^37^, who focused on examining the role of the socio-demographic factors, found age, sex, geographic location and socio-economic status all playing a role in the helmet usage frequency. Among the perceived barriers, the item indicating helmets suiting ones’ personal style had the highest negative correlation with helmet use, followed very closely by the items measuring discomfort according to Ledesma et al.^34^.

While the issue of helmet compliance is rather complex, its effectiveness has been confirmed by the current study. Høye^20,21^ notes that a higher helmet wearing rate is likely to be beneficial for all types of bicycle crashes (including single bicycle crashes). While there is no one definite solution to the injury prevention, the most successful measure will always depend on the geographical, cultural context and will likely involve multiple interventions working together. Molina-Soberanes et al.^38^ investigated cycling area as a confounder and effect modifier of the association between helmet use and cyclists’ risk of death after a crash and concluded an inverse relationship between cyclists’ helmet use and death. Historically, cycling safety has been explored using varied methodological approaches and from different angles. Framing of road safety in, for example journalism, has been also shown to play a role in reproducing assumptions about perceived risks, responsibilities, and role assignment of road users^39^.

Although the current study broadens the understanding of the effectiveness of bicycle helmets and evaluates the findings in the societally relevant contexts, the study has its limitation. First, because it has a strict review criterion it takes in consideration only a limited number of former meta-studies. Second, the studies included are not geographically representative and due to the data collection restrictions in some parts of the world, the availability of meta-analysis is scarce. Lastly, despite that fact that the authors attempted to investigate bicycle related crashes from multiple perspectives, the nature and the occurrence of cycling crashes and the role that helmet use plays is very complex, location specific and therefore difficult to capture.

Nevertheless, as urban planning moves from using oversimplified analogies derived from physics to becoming increasingly interdisciplinary and incorporating more insights from, for example, psychology, law, emergency medicine, the transportation field and its priorities may also continue to shift over time^33,40,41^. With respect to the societal costs, Gössling et al. ^42^ extrapolated that in the European Union, the external costs of automobiles are about 500 billion Euros per year, while cycling and walking make up benefits of 24 billion Euros and 66 billion Euros per year, therefore from a cost perspective, amplifying active travel and increasing the safety of cycling are worth investing in, particularly in the context of climate goals^43,44^.

## Data Availability

For the systematic review, the authors searched the following databases: SCImago Journal Rank https://www.scimagojr.com/journalrank.php, Transport Research International Documentation (TRID) database https://trid.trb.org/, Google Scholar https://scholar.google.com/, and Litmaps https://www.litmaps.com/. The datasets generated and/or analyzed during the current study are available from the corresponding author on reasonable request.
